# Map-Based Cloning and Functional Analysis of *YE1* in Rice, Which Is Involved in Light-Dependent Chlorophyll Biogenesis and Photoperiodic Flowering Pathway

**DOI:** 10.3390/ijms20030758

**Published:** 2019-02-11

**Authors:** Youlin Peng, Ting Zou, Lamei Li, Shiwen Tang, Qiao Li, Jie Zhang, Yongjun Chen, Xuechun Wang, Guotao Yang, Yungao Hu

**Affiliations:** 1 Rice Research Institute, Southwest University of Science and Technology, Mianyang 621010, China; youlinp@hotmail.com (Y.P.); xkdllm@126.com (L.L.); shiwentang_th@sina.com (S.T.); zhangjie0930123@163.com (J.Z.); cyj6495453@163.com (Y.C.); xuechunwang@swust.edu.cn (X.W.); yangguotao2377893@163.com (G.Y.); 2 College of Landscape Architectural, Sichuan Agricultural University, Chengdu 611130, China; lqsdswj@126.com

**Keywords:** rice (*Oryza sativa* L.), light, chlorophyll biosynthesis, photoperiodic flowering, map-based cloning

## Abstract

Light is one of the most important environmental factors that affect many aspects of plant growth, including chlorophyll (Chl) synthesis and flowering time. Here, we identified a rice mutant, *yellow leaf and early flowering* (*ye1*), and characterized the gene *YE1* by using a map-based cloning method. *YE1* encodes a heme oxygenase, which is localized to the chloroplasts. *YE1* is expressed in various green tissues, especially in leaves, with a diurnal-rhythmic expression pattern, and its transcripts is also induced by light during leaf-greening. The mutant displays decreased Chl contents with less and disorderly thylakoid lamellar layers in chloroplasts, which reduced the photosynthesis rate. The early flowering phenotype of *ye1* was not photoperiod-sensitive. Furthermore, the expression levels of Chl biosynthetic genes were downregulated in *ye1* seedlings during de-etiolation responses to light. We also found that rhythmic expression patterns of genes involved in photoperiodic flowering were altered in the mutant. Based on these results, we infer that *YE1* plays an important role in light-dependent Chl biogenesis as well as photoperiodic flowering pathway in rice.

## 1. Introduction

The growth of plants is affected by various environmental factors, including light, temperature and gravity. Among them, light has special and important significance for green flowering plants. Light is not only a source of energy for photosynthesis, but also an important signal for plant growth [1]. Light regulates several aspects of plant development—including germination, stem growth, leaf and root development, and phototropism—as well as Chl synthesis and flowering time [2]. 

Chl is an essential pigment involved in photosynthesis in chloroplasts [3]. It captures, transforms and redirects light energy [4]. These processes are critical for photosynthetic organisms. In higher plants, Chl contains chlorophyll *a* (Chl *a*) and chlorophyll *b* (Chl *b*). The Chl synthesis process starts from glutamate through a series of processes to produce Chl *a*, and then catalyzed by chlorophyllide *a* oxygenase (CAO) to form Chl *b* [5]. In angiosperms, the synthesis of Chl is inseparable from the participation of light [6]. In rice, mutants of *OsCAO1* gene have pale green leaves; and the expression of *OsCAO1* is induced by light [7]. 5-aminolevulinic acid (ALA) is a key precursor in chlorophyll biosynthesis [8]. Illumination increases the ALA accumulation; the expression levels of genes encoding glutamyl-tRNA reductase (GluTR) and glutamate-1-semialdehyde aminotransferase (GSAT) that catalyze ALA synthesis are also rapidly upregulated after illumination [9]. Phytochrome (PHY) mainly perceives red light and far red light [10]. In *Arabidopsis*, PHYA and PHYB together regulate the expression of *HEMA*, the gene encoding a GluTR for ALA synthesis [11,12]; phytochrome-interacting factor 1 (PIF1) directly or indirectly regulates the expression of *POR* (encoding a protochlorophyllide oxidoreductase) and *FeCh II* (encoding a ferrochelatase), which are key genes in Chl synthesis pathways [13].

Flowering is the conversion of vegetative growth to reproductive growth and is the most important process for determining the reproduction in angiosperms [14]. In addition to the Chl synthesis, light also regulates flowering time of plant through photoperiod [15]. Isolation of genes related to photoperiod response revealed the molecular mechanism about the flowering time in plants. In *Arabidopsis*, *CONSTANS* (*CO*) gene encodes a transcription factor that determines the integration of the circadian clock signal into light signal and regulates the transcript level of *FLOWERING LOCUS T* (*FT*), which induces flowering in response to long-day (LD) [16,17]. In the normal day/night cycle, the transcription of *CO* changes rhythmically within a day [18]. The expression of the *CYCLING DOF FACTORs* (*CDFs*) and *GIGANTEA* (*GI*) in the photoperiod pathway is regulated by the circadian rhythm [19,20]. CDFs and GI sense the circadian rhythm of clock genes and transmit signals to CO. *CO* expression is inhibited by *CDF* genes and is promoted by *GI*. In the afternoon, CDF protein is degraded by FLAVIN BINDING, KELCH REPEAT, F-BOX 1 (FKF1), thus the expression of CO will increase [19]. Under short-day (SD) conditions, the expression of *CO* only reaches a high level at night; under LD conditions, *CO* transcription can reach high level at dusk and night; this difference in expression of *CO* between LD and SD conditions ultimately determines the diversification in flowering time of plants [21,22]. Cryptochrome (CRY) and PHY also participate in the regulation of photoperiodic flowering [23]. PHY senses red light and far red light, while CRY perceives blue light and ultraviolet light. They regulate circadian rhythms by playing as a role of day-length and light strength sensors and stabilizing transcriptional products of *CO* [24].

Heme oxygenase (HO) converts the heme to biliverdin IX (BV), ferrous iron (Fe^2+^), and carbon monoxide (CO) [25]. BV is required for the synthesis of phytochrome-chromophore (PΦB) in plants [26]. In *Arabidopsis*, HO1 has been shown to be localized to the chloroplast; *hy1* mutant is caused by the mutation of *HO1* and may have defects in PΦB biosynthesis [27]. In maize, ELONGATED MESOCOTYL 2 (ELM2) is a HO1 homolog; *elm2* mutant seedlings have deficient Chl synthesis in de-etiolation responses to red light and far red light [28]. In rice, a homologous genes of *HO1* and *OsHO1* have been identified from rice nuclear genome. *OsHO1* is expressed in green tissues [29], with a higher expression level during the daytime and a lower expression level in the late afternoon and at night [30]. A knockdown mutant of *OsHO1* showed yellow-green leaves with defects in Chl biosynthesis [29]. Another allelic mutant with a 1-bp deletion in the first exon of *OsHO1* exhibits early flowering and photoperiodic insensitivity [30,31]. These findings suggest that *OsHO1* may participate in responses of Chl biogenesis or flowering control to light. 

In this study, we performed a map-based cloning of the *ye1* locus in rice and revealed that *ye1* harbors a single-base substitution in the coding region of *OsHO1*, which resulted in a premature stop codon in its translational products. Based on expression analysis of *YE1* and related genes and the phenotypic observation of *ye1*, we proposed that *YE1* plays important roles in both two pathways of light-dependent chlorophyll biogenesis and photoperiodic flowering at the same time.

## 2. Results

### 2.1. ye1 Mutant Exhibits Impaired Chloroplast Development and Photosynthesis

**Figure 1:**
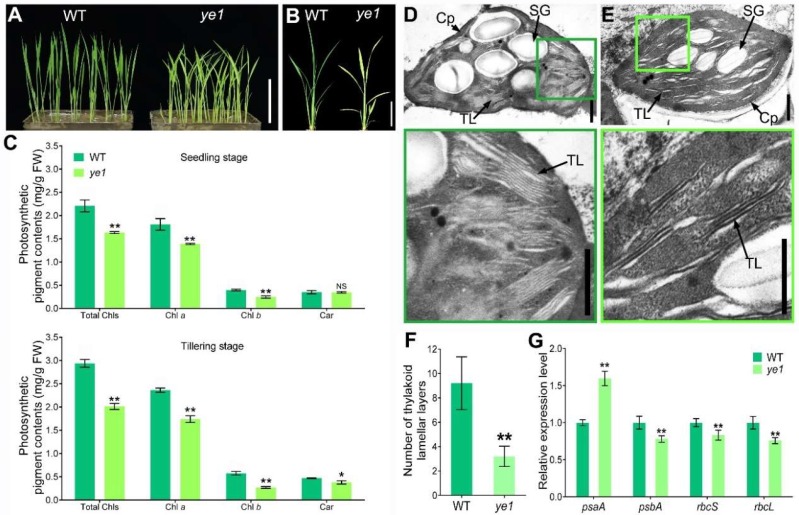
Leaf-color phenotypes of WT and *ye1*. (**A**) The appearance of WT and *ye1* at the seedling stage. Bar = 10 cm. (**B**) The appearance of WT and *ye1* at the early tillering stage. Bar = 10 cm (**C**) Comparisons between the WT and *yel* in pigment contents at the seedling stage and tillering stage. (**D**,**E**) TEM analysis of leaves in the WT (**D**) and *yel* (**E**) seedlings. Cp, chloroplast; SG, starch grain; TL, thylakoid lamellar layer. Bars = 1 μm. (**F**) Number of thylakoid lamellar layers in the chloroplasts of WT and *ye1* seedlings. (**G**) Expression levels of genes related to Photosynthesis in WT and *yel* plant at the heading stage. Each value is given as means ± standard deviation (SD) or standard errors of mean (SEM) (*n* = 3). NS, no significant change. * (*p* < 0.05) and ** (*p* < 0.01) indicate significant differences between WT and *ye1* (Student’s *t*-test).

The *ye1* mutant was isolated from a rice mutant library (in background of a *Japonica* cultivar Wuyungeng 21) mutagenized with ethyl methanesulfonate (EMS). At the seedling stage, *ye1* showed a yellow leaf phenotype, while the leaves of the wild-type Wuyungeng 21 (WT) appeared pale-green (Figure 1A). Compared with the green leaves of the WT, *ye1′*s leaves were yellow-green at the early tillering stage (Figure 1B). To explore the yellow leaf phenotype of *ye1* further, we measured the photosynthetic pigment contents at two different stages. The results showed that, at the seedling stage, *ye1* had significant reductions of Chl *a*, Chl *b*, and total Chls, whereas the carotenoids were comparable compared with those in the WT (Figure 1C). At the tillering stage, although the Chl contents of *ye1* is increased compared with which at the seedling stage (Figure 1C), they were still significantly lesser than that of the WT (Figure 1C). Besides, the level of carotenoids in *ye1* decreased at the tillering stage compared with that of the WT (Figure 1C). These data suggested that leaf-color phenotype of *ye1* was caused by the reduced Chl contents. 

To investigate the chloroplast development in *ye1*, transmission electron microscope (TEM) analysis was performed to compare the ultrastructure of chloroplasts in the leaves of WT and *ye1* at the seedling stage. Although the mesophyll cells of WT (Figure 1D) and *ye1* (Figure 1E) displayed fully developed chloroplasts, the decreased number of thylakoid lamellar layers was observed in *ye1*, and these thylakoid lamellar layers were poorly arranged compared with which of the WT (Figure 1D–F). These results suggested that *ye1* had impaired chloroplast development that correlated with its leaf-color and Chl contents. To determine whether photosynthetic apparatus was affected in *ye1*, we examined the parameters of Chl fluorescent and photosynthetic efficiency between the WT and *ye1* under field conditions. As shown in Table 1, significant reductions of the effective quantum yield of PSII (ΦPSII), electron transport rate (ETR), and maximum quantum yield of PSII (Fv/Fm) were detected in *ye1* compared with the WT. Moreover, *ye1* had much lower values in photosynthetic efficiency of net photosynthetic rate (Pn), stomatal conductance (Gs), and transpiration rate (Tr) than those of the WT, while changes in intercellular CO_2_ concentration (Ci) were not evident (Table 1). The expression levels of photosynthesis-associated genes of the WT and *ye1* were also detected using real-time quantitative PCR (qPCR). The results showed that the transcript levels of a gene encoding the subunit of photosystem I (*psaA*) was significantly upregulated in *ye1*, whereas genes encoding the core component of Photosystem II (*psbA*), small subunit of Rubisco (*rbcS*) and Rubisco large subunit (*rbcL*) apparently downregulated in *ye1* compared with those of the WT (Figure 1G). These findings suggested that photosynthesis was deficient in *ye1*.

### 2.2. ye1 Mutant Displays Early Flowering

**Figure 2:**
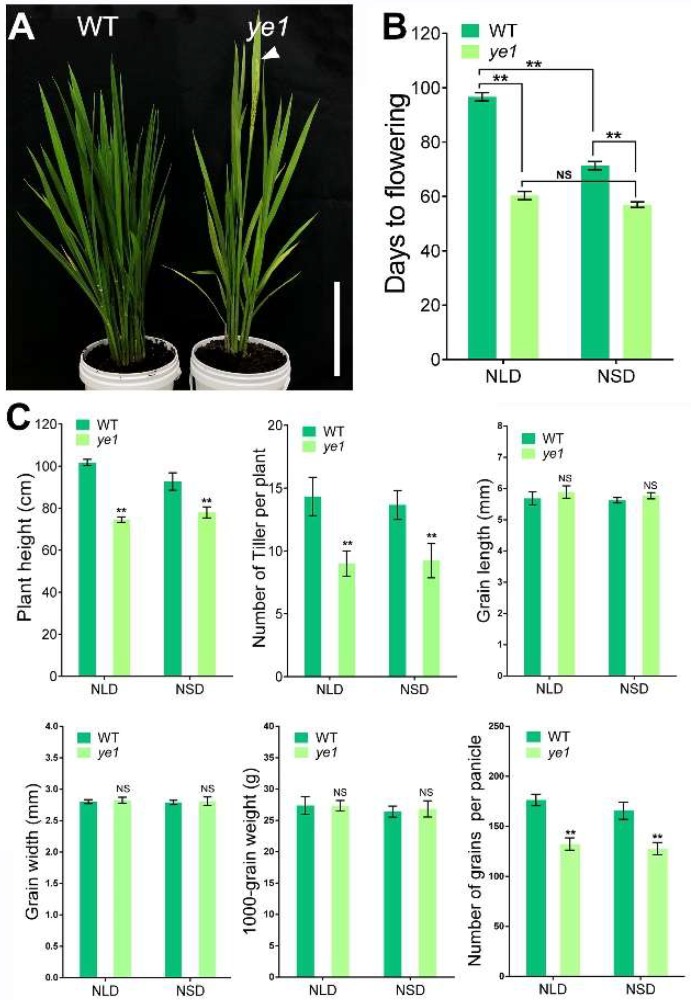
Flowering time phenotypes and other agronomic traits of WT and *ye1*. (**A**) Phenotype of WT and *ye1* under NLDs, when *ye1* was heading. The arrow indicates the flower. Bar = 25 cm. (**B**) Days to flowering of WT and *ye1* under NLDs and NSDs (mean ± SD, *n* = 3). NS, no significant change. Significant differences were shown as ** (*p* < 0.01) by Student’s *t*-test (**C**) Other agronomic traits (including plant height, tillering number, grain length, grain width, 1000-grain weight, and number of grains per panicle) of WT and *ye1* under NLDs and NSDs. Each value is given as means ± SD (*n* = 3). NS, no significant change. ** (*p* < 0.01) indicate significant differences between WT and *ye1* (Student’s *t*-test).

In addition to the yellow leaf, the *ye1* mutant was found to have an early flowering phenotype (Figure 2A). Under natural LD (NLD) condition, *ye1* showed a 36.4-day earlier flowering than the WT (Figure 2B). Moreover, the number of days to flowering (DTF) of *ye1* also reduced by 14.3 under natural SD (NSD) condition compared with the WT (Figure 2B). Although a significant decrease was found in the DTF of the WT under NSD condition when compared with which under NLD condition, *ye1* showed a similar DTF under these two different photoperiodic conditions (Figure 2B). These results indicated that the early flowering phenotype of *ye1* was not photoperiod-sensitive.

The other agronomic traits of WT and *ye1* were also compared under NLD or NSD condition. In contrast to the WT, the main agronomic traits including plant height, number of tillers per plant, and number of grains per panicle were remarkably reduced in *ye1* under NLD or NSD conditions (Figure 2C and Figure S1). However, no significant difference in grain size and weight was found between the WT and *ye1* (Figure 2C).

### 2.3. Map-Based Cloning of YE1 and Complementation Analysis

**Figure 3:**
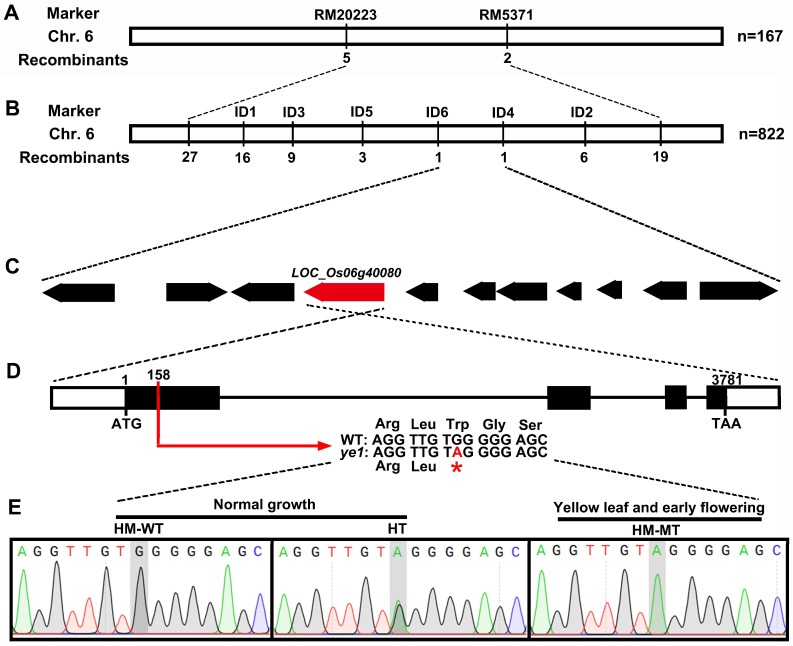
Map-based cloning and identification of the *YE1* gene. (**A**) The YE1 locus was mapped to the interval between molecular markers RM20223 and RM5371 on chromosome 6. (**B**) The YE1 locus was further delimited to a ~85 kb genomic region between markers ID4 and ID6. (**C**) The candidate region contained eleven ORFs. (**D**) The gene structure of candidate LOC_06g40080. The red arrow indicates the ye1 mutation at 158 nucleotide position in *LOC_06g40080*, which changes Ter at position 53 to a stop codon. The asterisk indicates the stop codon. (**E**) The identification of the *YE1* gene in the ye1/WT F_2_ population by sequencing.

For genetic analysis of the *ye1* mutant, cross between *ye1* and *indica* variety Shuhui 498 was generated. All F_1_ plants displayed normal green leaves and heading dates. Among the 4163 individuals in F_2_ population, 1062 plants showed the phenotype of yellow leaves and early flowering, whereas the other plants of F_2_ population grew normally. These data indicated that the separate ratio of F_2_ plants was about 3:1 (3101:1062, χ^2^ = 0.5785 < χ^2^_0.05_ = 3.84), suggesting that the abnormal leaf-color and flowering time phenotype of *ye1* was controlled by a single recessive nuclear locus. We then performed map-based cloning to identify the YE1 locus. The YE1 locus was initially mapped to a region on the long arm of chromosome 6 between SSR markers RM20223 and RM5371 (Figure 3A). To fine map the YE1 locus, we developed several polymorphic InDel (ID) markers between ye1 and Shuhui 498. By using these ID markers to analyze 822 individuals with yellow leaves and early flowering, the YE1 locus was delimited to an 84.7 kb interval between ID4 and ID6 (Figure 3B). Within the fine mapping region, a total of 11 open reading frames (ORFs) were predicted (http://rice.plantbiology.msu.edu, Figure 3C). We then sequenced the genome DNA of these ORFs. Among them, only one single nucleotide polymorphism (SNP) mutation located in the first exon of *LOC_Os06g40080* was found, compared with which of the WT (Figure 3D). This mutation yields a premature stop codon (Figure 3D) and was predicted to produce truncated polypeptides that resulted in a deletion of the whole HO domain (PFAM Accession Number: PF01126.13, Figure S2). Further linkage analysis was carried out by sequencing the mutation site of individuals from the F2 population generated from the cross between *ye1* and WT. The results showed that all of the plants with yellow leaves and early flowering had the same homozygous mutation at this locus, whereas the normal plants were homozygous wild-type or heterozygous genotype, indicating this mutation was co-segregated with the leaf-color and flowering time phenotype of *ye1* (Figure 3E and Table S1). 

**Figure 4:**
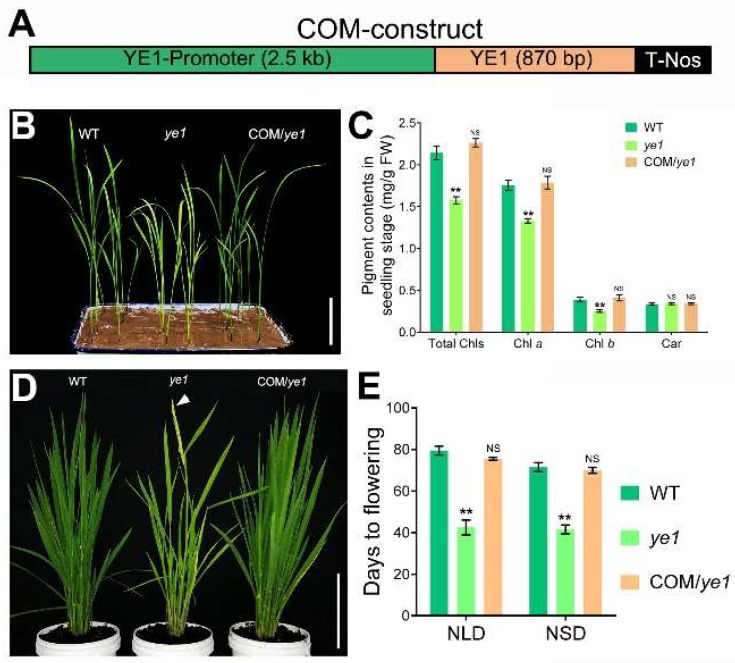
Complementation of *ye1* mutant. (**A**) The construct for complementary analysis of *YE1* gene. (**B**) Leaf color phenotypes of WT, *ye1* and complementary plants (COM/*ye1*). Bar = 5 cm. (**C**) Comparisons of pigment contents of WT, *ye1* and complementary plants. (**D**) Flowering time phenotypes of WT, *ye1* and complementary plants under NLDs, when *ye1* was heading. The arrow indicates the flower. Bar = 25 cm. (**E**) Days to flowering of WT, *ye1* and complementary plants under NLDs and NSDs. Each value is given as means ± SD (*n* = 3). NS, no significant change. * (*p* < 0.05) and ** (*p* < 0.01) indicate significant differences compared with WT (Student’s *t*-test).

To confirm whether *LOC_Os06g40080* corresponds to *YE1*, we produced transgenic rice plants by introducing a construct consisting of a 2.5-kb upstream promoter and the full-length cDNA sequence of the wild-type *LOC_Os06g40080* gene (Figure 4A) to the *ye1* mutant via *Agrobacterium*-mediated transformation. Phenotypic observations showed that all the positive transgenic lines had normal green leaves (Figure 4B); and their photosynthetic pigment contents were also indistinguishable from that of the WT (Figure 4C). Besides, the number of DTF of these positive transgenic lines were similar to that of the WT under NLD or NSD condition (Figure 4D,E). Taken together, the results suggested that *LOC_Os06g40080* is the *YE1* gene, and that *ye1* is a loss-of-function mutant of *YE1* gene.

### 2.4. YE1 Protein Is Conserved in Land Plants

**Figure 5:**
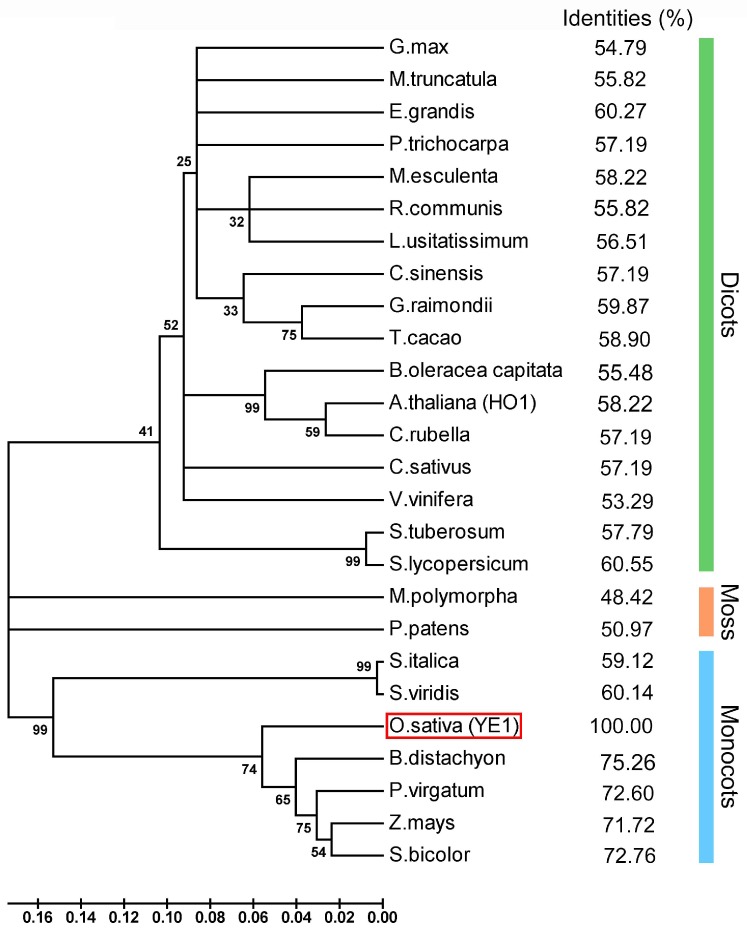
Protein phylogenic analysis among YE1 and YE1-related proteins. A neighbor-joining phylogenic analysis was performed using MEGA 5.0, based on the alignment result in Supplementary Figure S3. The YE1-related proteins are clearly clustered into three clades. Green, orange and blue bars represent the three different clades, which indicate the homologous proteins of YE1 from the species in dicots, moss and monocots, respectively. The YE1 protein is highlighted by the red box. The numbers at the nodes indicate the bootstrap value. The percentage numbers represent the identities of corresponding proteins to YE1.

*YE1* (*LOC_Os06g40080*) was annotated to encode HO1 in rice (OsHO1, http://rice.plantbiology.msu.edu). This protein was reported previously as the key regulator of photoperiod sensitivity (named SE5) or chlorophyll biosynthesis (named YGL2) [29,30,31]. In this study, we refer to OsHO1/SE5/YGL2 as ‘YE1′. By using YE1 protein sequence as a search query for BLASTP in the Phytozome database (http://www.phytozome.net), 25 closest related proteins of YE1 from other land plant species were retrieved (Table S2). Multiple peptides alignment results of these proteins indicated that their HO domain were highly conserved among different species, whereas a high sequence diversity was found in their N-terminal region (Figure S3). A neighbor-joining phylogenetic tree of these proteins were constructed based on the results of protein sequences alignment. The YE1-realtives were clustered into three clades-dicots, mosses and monocots, the proteins in which had ~57%, ~50%, and ~73% identity to the YE1 protein, respectively (Figure 5). These results suggested that YE1 is conserved in land plants, and may share similar functions with its homologs in other species.

### 2.5. Expression Pattern and Subcellular Location of YE1

**Figure 6:**
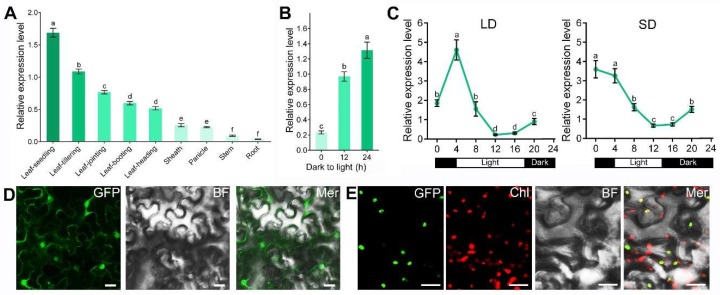
The expression pattern and subcellular localization of YE1. (**A**) *YE1* expression levels in different tissues. (**B**) *YE1* expression levels during light-induced greening of WT etiolated seedlings. After growing in darkness for 10 days, etiolated WT seedlings were illuminated for 12 or 24 h. (**C**) Rhythmic expression of *YE1* under LDs and SDs. (**D**) Subcellular localization of GFP protein (control). (**E**) Subcellular localization of YE1–GFP fusion protein. BF, bright field; Chl, chloroplasts’ auto-fluorescence; Mer, merge of each channel. Bars = 10 μm. Each value is given as means ± SEM (*n* = 3). Different letters indicate the statistical differences at *p* < 0.05 by Duncan’s test.

The expression pattern of *YE1* gene was detected using qPCR. The results showed that *YE1* exhibited a strong expression in leaf, with a relatively weaker mRNA level in root, stem, sheath, and panicle (Figure 6A). Besides, the maximal transcription level of *YE1* was observed within the leaf at the seedling stage, but its expression extremely reduced at tillering, jointing, and booting stage, and was relatively weak at heading stage, suggesting that expression of *YE1* was also influenced by leaf developmental stage. To test whether *YE1* expression is regulated by light, we examined the temporal expression patterns of *YE1* during leaf greening of the WT etiolated seedlings. The results showed a sharply increased expression of *YE1* after exposure of 10-day-old etiolated WT seedlings to light (Figure 6B), suggesting that transcription of *YE1* is induced by light during leaf greening. Due to the mutation of *YE1* caused a photoperiod-insensitive phenotype of *ye1*, we therefore tested the behavior of *YE1* in the WT plants under different photoperiodic conditions. Our qPCR results showed that the transcripts of *YE1* started to accumulate from 8 h after dawn, reaching a peak at 10 h after dusk under both LDs and SDs (Figure 6C), suggesting that the expression of *YE1* is diurnal-rhythmic.

To determine the subcellular localization of YE1 protein, we fused green fluorescent protein (GFP) to the C-terminal of YE1 under the control of the cauliflower mosaic virus 35S promoter (*p35s::YE1-GFP*), and transiently expressed this plasmid in the epidermal cells of tobacco leaves. Transient expression results showed that, as the control, the *p35s::GFP* were expressed throughout the cells (Figure 6D), whereas the strong fluorescent signals of *p35s::YE1-GFP* were observed in the chloroplast-like structures of the cells (Figure S4). To confirm this subcellular localization pattern, we observed the auto-fluorescence of chloroplasts. Via merging the micrographs from each channel, we found that the detected green fluorescent signals of *p35s::YE1-GFP* completely overlapped with chloroplasts’ auto-fluorescence (Figure 6E). This result suggested that YE1 protein is targeted to the chloroplasts.

### 2.6. YE1 Is Involved in Light-Dependent Chl Synthesis

**Figure 7:**
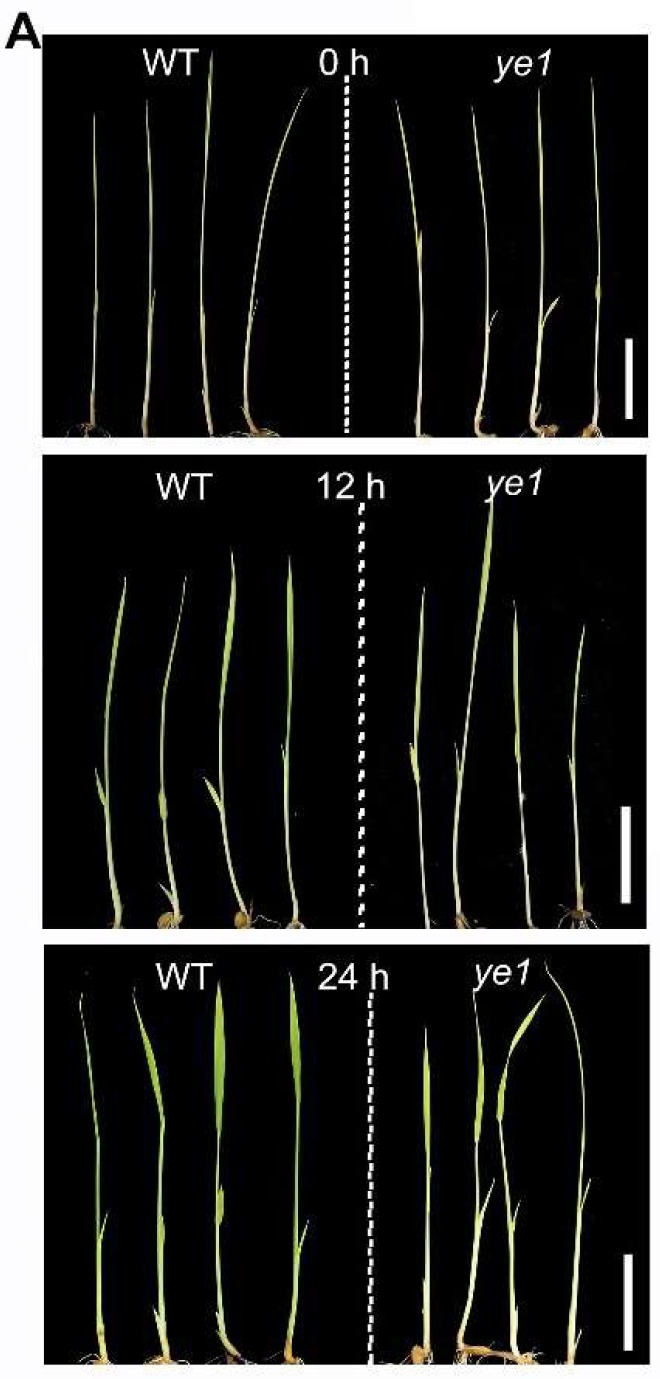

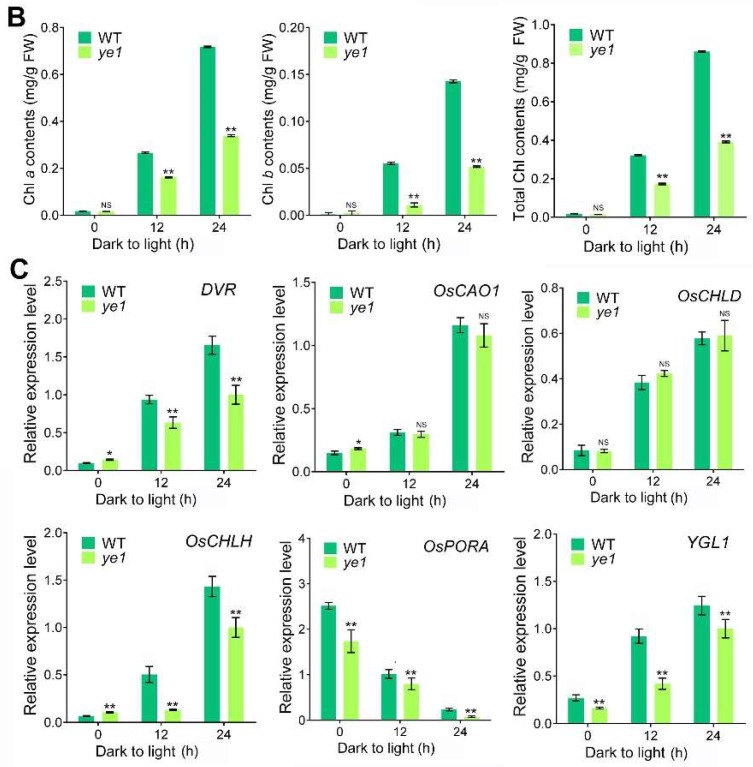
Expression levels of Chl synthesis-associated genes in WT and *ye1*. (**A**) Comparisons of greening speed in WT and *ye1* etiolated seedlings that were exposed to light for 12 or 24 h. Bar = 5 cm. (**B**) Comparisons of Chl contents in the WT and *ye1* etiolated seedlings during light-induced greening. (**C**) Comparisons of changes in expression levels of Chl synthesis-associated genes in WT and *ye1* etiolated seedlings during light-induced greening. Each value is given as means ± SD or SEM (*n* = 3). NS, no significant change. * (*p* < 0.05) and ** (*p* < 0.01) indicate significant differences compared with WT (Student’s *t*-test).

The synthesis of Chl molecules is strongly induced by light exposure in angiosperms [6]. To explore the contribution of *YE1* to light-dependent Chl synthesis, we compared the Chl contents of the WT and *ye1* etiolated seedlings during light-induced leaf greening. Unlike the WT etiolated seedlings that quickly turn green after being exposed to light, *ye1* seedlings remained pale yellow after 24 h of illumination (Figure 7A). The accumulation of both Chl *a* and Chl *b* in *ye1* plants lagged far behind ones in the WT plants throughout the whole light exposure period (Figure 7B). To understand the molecular basis of *YE1* involvement in light-dependent Chl synthesis, we also examined the temporal expression profiles of several Chl synthesis-associated genes during light-induced leaf greening of the WT and *ye1* etiolated seedlings. For example, *DVR* encodes a divinyl reductase, which catalyze the conversion of divinyl Chl *a* to monovinyl Chl *a* [32,33]; *OsCAO1* encodes a Chl *a* oxygenase, which catalyze the synthesis of Chl *b* from Chl *a* [7,34]; *OsCHLH* and *OsCHLD* participate in the synthesis of Chl precursors by producing two different subunits of magnesium-chelatase [35,36,37,38,39,40]; OsPORA is a NADPH: protochlorophyllide oxidoreductase A in rice, and catalyzes the photoreduction of chlorophyllide from protochlorophyllide in Chl synthesis [41]; *YGL1* encodes a Chl synthase that catalyzes the esterification of chlorophyllide to form Chl *a* [42]. The qPCR results showed that (Figure 7C), the transcription levels of *DVR*, *OsCAO1*, *OsCHLD*, *OsCHLH*, and *YGL1* were upregulated during light-induced greening in WT seedlings, suggesting that these genes are required for the light-dependent Chl synthesis during de-etiolation. Consistent with the previous report [41], *OsPORA* expression drastically decreased upon illumination. However, compared with that in the WT plants, the expression of *DVR*, *OsCHLH*, *OsPORA*, and *YGL1* were reduced in *ye1* plants. Only *OsCAO1* and *OsCHLD* expression levels in *ye1* were comparable with that of the WT. These observations indicated that the transcript levels of genes related to Chl synthesis were altered in *ye1* during leaf greening and suggested that *YE1* likely plays an important role in the light-dependent synthesis of Chl during de-etiolation.

### 2.7. Diurnal Expression Patterns of Flowering Genes Are Altered in ye1

**Figure 8:**
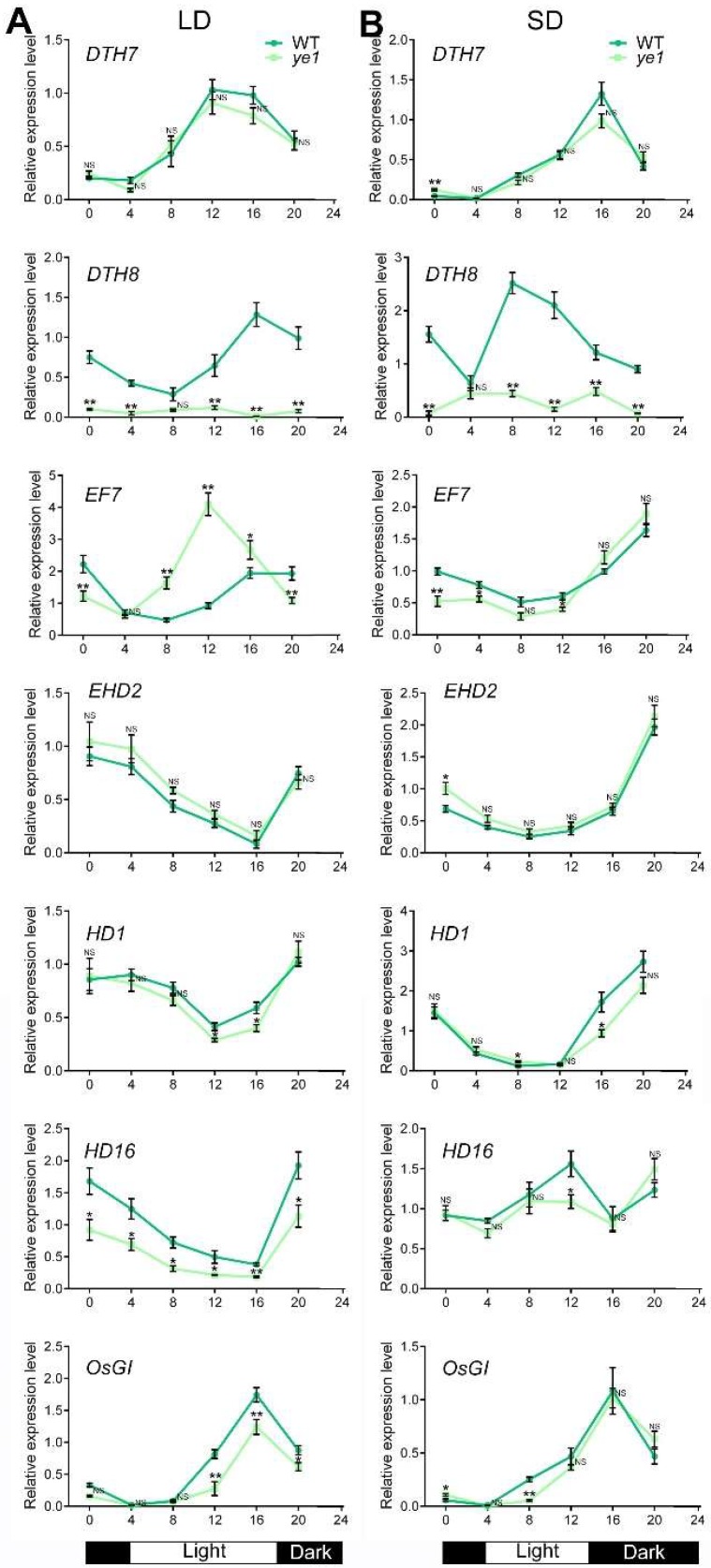
Rhythmic expression patterns of photoperiodic flowering-associated genes in WT and *ye1*. (**A**) Rhythmic expression patterns of *DTH7*, *DTH8*, *EF7*, *EHD2*, *HD1*, *HD16*, and *OsGI* under LDs. (**B**) Rhythmic expression patterns of *DTH7*, *DTH8*, *EF7*, *EHD2*, *HD1*, *HD16*, and *OsGI* under SDs. Each value is given as means ± SEM (*n* = 3). NS, no significant change. * (*p* < 0.05) and ** (*p* < 0.01) indicate significant differences compared with WT (Student’s *t*-test).

The photoperiodic pathway is one of the most important pathways for plants flowering control. Several genes have been reported to be involved in rice photoperiodic flowering pathway. For example, *Days to heading* (*DTH*) *7* is a major genetic locus that controls the delayed flowering of rice under LDs, and its expression is regulated by photoperiod [43]. DTH8 encodes a HAP3 subunit of HAP complex, and delays flowering by inhibiting the expression of ‘florigen’ genes under LDs [44]. *Early flowering 7* (*EF7*) gene is a rice ortholog of *Arabidopsis ELF3*, which promotes flowering under both LDs and SDs [45]. *Early heading date 2* (*EHD2*) is a key factor in the flowering transformation of rice and encodes a transcription factor with a zinc finger motif; it is an ortholog of INDETERMINATE1 (ID1), which also promotes flowering in maize [46,47]. Heading date 1 (HD1) is a member of the rice CO family, which promotes flowering under SDs but inhibits flowering under LDs; *HD1* is located downstream of *EHD2*, and is an important integration point of signaling in the conserved pathway of flowering regulation [47,48,49]. *Heading date 16* (*Hd16*) encodes a casein kinase I that inhibits flowering under LDs [50]. The expression of *OsGI* is controlled by photoperiod, which is similar to its homolog in *Arabidopsis*; but under LDs, *OsGI* inhibits flowering, which is different from *Arabidopsis GI* [51,52,53]. We therefore compared the expression levels of these genes between the WT and *ye1* mutant under different photoperiodic conditions. qPCR results indicated that the peak phases of *DTH7* expression slightly decreased in *ye1* mutants under both LDs (Figure 8A) and SDs (Figure 8B) compared with which of the WT. Similarly, the expression levels of *HD16* and *OsGI* in *ye1* mutant were lower than that of the WT under LDs (Figure 8A). However, mRNA levels of *EHD2* were not significantly affected in *ye1* mutant (Figure 8A,B). Transcript levels of *DTH8* were largely reduced in *ye1* mutants under both LDs (Figure 8A) and SDs (Figure 8B). Strikingly, under LDs, but not SDs, *EF7* mRNA levels were markedly upregulated in *ye1* mutants after dawn (Figure 8A). These results suggested that the mutation in *YE1* affected the expression profiles of the genes known to be related to photoperiodic flowering.

## 3. Discussion

Rice is one of the most important food crops in the world, and is also a model of monocots. To date, a range of genes in rice have been reported to function in Chl biogenesis, such as *DVR*, *FGL*, *LYL1*, *OsCAO1*, *OsCHLD*, *OsCHLH*, *OsPORA*, *OsPORB*, *YGL1*, *YGL2*, and *YLC1* [7,29,32,36,37,38,40,41,54,55,56,57]. Leaf Chl contents affect grain formation by affecting photosynthetic efficiency in rice [58]. Meanwhile, photoperiodic flowering has been extensively studied in rice, and many corresponding genes have been identified during past years, such as *DTH7*, *DTH8*, *EF7*, *EHD1*, *HD1*, *HD3A*, *OsGI*, *OsMADS50*, *OsMADS51*, *OsMADS56*, *RID1*, *RFT1*, and *SE5* [30,43,44,45,46,48,51,52,53,59,60,61,62,63]. In addition, flowering time is usually associated with vegetative growth as well as yield-related traits [64]. In this work, we isolate a rice mutant *ye1*. The *ye1* mutant has yellow leaves with a significant reduction of Chl content and impaired chloroplast development and photosynthesis (Figure 1), and shows early flowering phenotype (Figure 2A,B). Besides, in *ye1* mutant, plant height, tiller numbers, and grain numbers per panicle are also drastically decreased (Figure 2C), suggesting a low yield. Further investigations found that *YE1* is responsible for the phenotypes of *ye1*, suggesting that mutation in *YE1* results in low yields, and *YE1* may have potential application of improving yields in rice breeding.

Light response plays an important role in plant growth, development, and metabolism. Light induces Chl synthesis in the de-etiolation process of plant seedlings [2]. The results of this work show that the expression of *YE1* gene peaks in WT seedling leaves (Figure 6A) and is induced by light during leaf greening (Figure 6B). Moreover, Chl synthesis in *ye1* etiolated seedlings lags far behind that of WT (Figure 7A and B), suggesting that *YE1* is involved in light-dependent Chl synthesis. DVR, OsCAO1, OsCHLH, and YGL1 proteins were reported to be critical for Chl synthesis, and are encoded in the rice nuclear genome [7,32,38,42]. Our qPCR results indicated that transcript levels of these nuclear genes increased in WT plants after illumination (Figure 7C), which is consistent with previous reports [7], and suggest these genes may function in light-induced synthesis of Chl. However, *DVR*, *OsCHLH*, and *YGL1* expression were downregulated in the *ye1* plants (Figure 7C). This may cause the resulting lower Chl contents in *ye1* (Figure 7B). Though YE1 protein is located to the chloroplasts (Figure 6E), signals from the plastids to the nucleus is critical to regulate nuclear gene expression [65]. Based on these results, we speculate that YE1 may play an essential role in light-dependent Chl synthesis of chloroplasts and propose that YE1 may affect nuclear gene expression through plastid-to-nucleus retrograde signaling. 

In addition, light also affects the flowering time of plants through different photoperiods [2]. In this study, we show that *YE1* has different diurnal rhythmic expression patterns when under different day-length conditions (Figure 6C), suggesting that *YE1* may respond to the changes of photoperiods. Rice is a SD plant [51,66]. Similarly, in WT plants, DTF under NLDs is longer than which under NSDs (Figure 2B). However, flowering times of *ye1* mutant under between NLDs and NSDs are comparable (Figure 2B), suggesting that *ye1* mutant has less or no photoperiodic-sensitivity, and *YE1* may play roles in photoperiod response. This photoperiodic-insensitive phenotype of *ye1* is different from that of *hy1* mutant in *Arabidopsis* [67], whereas YE1 is an ortholog of *Arabidopsis* HO1 (Figure 5 and Figure S3), reflecting various roles of HO between monocots and dicots. Besides, the flowering time of our *ye1* mutant was significantly earlier than that of WT under NLDs (Figure 2A,B). Coincidently, expression analyses showed that transcript level of *EF7*, which promotes flowering both under LDs and under SDs [45], is significantly upregulated in *ye1* mutant under LDs but not obviously changed under SDs compared with which of WT (Figure 8). Expression of *DTH7*, *HD16*, and *OsGI*, which play roles in delaying flowering time under LDs [43,50,52], is suppressed in mutant under LDs (Figure 8A). Apart from that, *DTH8* is another major genetic factor that delays flowering by inhibiting the expression of ‘florigen’ genes under LDs [44]; its transcription decreased markedly in *ye1* mutant under both LDs and SDs (Figure 8). Collectively, these data suggest that *YE1* may participate in photoperiodic flowering control by affecting the diurnal expression profiles of genes involved in the photoperiod pathway.

In higher plants, phytochrome is a family of photoreceptors that receive and respond to red/far-red light [68], and also plays a major role in response to photoperiod [21,69]. PΦB participates in the regulation of many essential developmental processes (including seedling de-etiolation and flowering) through forming phytochrome by combining with apoprotein [70,71]. BV, as a product derived from HO-catalyzed oxidative cleavage of heme, is the precursor of PΦB [25,72]. In *Arabidopsis*, the mutation of *HO1* causes the *hy1* mutant to do not respond to red and far-red light [73], and be deficient in BV and subsequent PΦB synthesis [27]. Another HO-deficient mutant of pea (*pcd1*) was also shown to be unable to convert heme to BV and have severely impaired ability in PΦB biosynthesis [74]. These findings suggest that HO functions in PΦB biosynthesis, and is required for normal photomorphogenesis. Apart from affecting the plant’s response to light through producing BV, the substrate of the HO-catalyzed reaction may also be involved in the synthesis of Chl. The synthesis of ALA, which is catalyzed by GluTR and GSAT, is a key step in Chl biogenesis [75]. Thus, the enzymatic activity of GluTR is critical for Chl biosynthesis [76]. It was reported that application of exogenous heme reduced the enzymatic activity of barley GIuTR1 by 50% [77]. Both mutations in the *yg-2* and *au* mutants of tomato led to a yellow-green phenotype, and the normal heme turnover in the plastids of these mutants was blocked [78]. *Arabidopsis ulf3* is an allelic mutant of *hy1*. In *ulf3* mutant, the decrease in HO activity caused the accumulation of heme, while the accumulation of heme inhibited the activity of GluTR [79]. These data indicate that HO may participate in the biosynthesis of Chl by affecting the heme accumulation, which has feedback inhibition of the enzymatic activity of GluTR. Our results of peptides alignment indicated that the whole HO domain of YE1 in our *ye1* mutant is deleted (Figure S2), and YE1 is conserved in land plants (Figure 5). Combining the above findings with these results, we raise a possibility that *ye1* is likely to be a HO-deficient mutant, and may also have probable deficiencies of PΦB biosynthesis and GluTR activity, which may result in the mutant phenotypes of photoperiodic-insensitive flowering and impaired Chl biogenesis. 

The *se5* mutants display a serve defect in photoperiodic response and a very early heading date [31]. The *ygl2* is an allelic mutant of *se5*, and shows yellow-green leaves and a reduction of Chl contents [29], suggesting that *SE5*/*YGL2*, which encodes the OsHO1, may be related to Chl biosynthesis or photoperiodic flowering control. However, it is still unclear whether OsHO1 can participate in these two pathways at the same time. In the present study, our *ye1* mutant exhibits defective chloroplast development and photoperiodic response simultaneously. Results of map-based cloning revealed that *YE1* is another allele of *SE5*/*YGL2* but has different mutation site. The *se5* mutant was isolated from *japonica* rice cultivar Norin 8, and a single base deletion of A406 was found in its first exon, which caused the frame-shift of SE5 [31]. The *ygl2* mutant was derived from *indica* rice variety Gang 46B, and has a 7-kb insertion in the first exon, resulting in a decreased expression of *YGL2*. Our *ye1* mutant was characterized from *japonica* rice cultivar Wuyungeng 21. Sequencing results indicated a single base substitution from G158 to A158 at the first exon of *YE1* in *ye1* mutant. This point mutation changed a tryptophan (Trp) to a stop codon at the 53rd amino acid (Figure 3D), which resulted in the absence of the whole HO domain (Figure S2). Interestingly, *se5* and *ye1* exhibited the similar photoperiodic-insensitive flowering phenotype, whereas *ygl2* and *ye1* appeared similar defects in Chl biogenesis that has not been reported in *se5*. These findings suggest that YE1 may play roles in both Chl biogenesis and photoperiodic response, and we also infer that different mutation sites in various backgrounds may result in different manifestations of gene functions.

## 4. Materials and Methods

### 4.1. Plant Materials and Growth Condition

The mutant line *ye1* was derived from the *japonica* rice cultivar Wuyungeng 21 through EMS mutagenesis. EMS treatment and mutant screening were performed as described previously [80]. For phenotypic analysis, rice plants were grown and maintained regularly under either NLDs in Mianyang, Sichuan, China (average day-length >13.6) or NSDs in Lingshui, Hainan, China (average day-length <11.7). For light-dependent leaf greening experiments, rice seedlings were grown in continuous darkness for 10 days and subsequently exposed to light for 12 or 24 h. For diurnal rhythmic expression analysis, rice seedlings were cultivated in a growth chamber under either LDs (14 h of light/10 h of dark) or SDs (10 h of light/14 h of dark) for four weeks; leaves were then collected every 4 h, for 24 h.

### 4.2. Morphological, Physiological, and Cellular Analysis

Determinations of pigment contents and photosynthetic and Chl fluorescence parameters for leaves were performed as described previously [81]. To observe the chloroplasts of seedling leaves, a TEM (Hitachi, H-600, Tokyo, Japan) was employed, and the samples were prepared as described previously [82]. The grain length, width, and 1000-grain weight were measured using an automatic seed-size analyzing system (Wanshen, SC-G, Shenzhen, China). Other agronomic traits were investigated using conventional methods at maturing stage. The data were analyzed using the software program GraphPad Prism 7.0 (https://www.graphpad.com/, San Diego, CA, USA) for mean values and SD or SEM. Statistical significance was assessed by conducting Student’s *t*-test or Duncan’s test. 

### 4.3. Map-Based Cloning of YE1

The *ye1* mutant was crossed with an *indica* variety Shuhui 498, and the resulting F1 was further selfed to generate the F2 population. For primary mapping, 167 F_2_ mutant plants were used to map the YE1 locus to the region between markers RM20223 and RM5371 on chromosome 6. The YE1 locus was further fine mapped to an 84.7-kb genomic interval between the markers ID6 and ID4 by using 822 F_2_ mutant plants. The mutation of *YE1* gene in the mutant was confirmed by PCR products sequencing with the primer set YE1-SEQ. All the primers used in this study are listed in Table S3.

### 4.4. Vector Construction and Transgenic Analysis

To verify the *YE1* function, we generated a complementary construct by amplifying the full-length *YE1* cDNA and its native promoter (a 2.5-kb DNA fragment upstream of the start codon ATG of *YE1*) from WT with primer sets YE1-COM and PYE1-COM, respectively. These two fragments were thereafter inserted into the plant binary vector pCAMBIA1300 in front of NOS terminator to generate the COM-construct (*pYE1::YE1*). The COM-construct was introduced into the *Agrobacterium tumefaciens* strain EHA105, and was then transferred into the mutant in accordance with a method published previously [83]. Eight positive transgenic lines were obtained.

### 4.5. Expression Analysis

Total RNA of various rice tissues was extracted using the E.Z.N.A.^®^ Plant RNA Kit (Omega Bio-tek, Norcross, GA, USA) and reverse transcribed using the HiScript^®^ Q Select RT SuperMix kit (Vazyme, Nanjing, China) in accordance with the manufacturer’s instructions. qPCR was performed by using the AceQ qPCR SYBR Green Master Mix (Vazyme, Nanjing, China) with the corresponding primer sets on a Real-Time PCR System (Bio-Rad, CFX96, Hercules, CA, USA). *OsACTIN1* was used as the normalized reference gene. The relative levels of gene expression were calculated as described previously [84].

### 4.6. Subcellular Localization of YE1

To determine the subcellular localization of YE1, the full-length cDNA of *YE1* was amplified with primer set YE1-GFP, and was then cloned into the expression vector pBI121-GFP, resulting in a C-terminal fusion with GFP. This plasmid was expressed in tobacco (*Nicotiana benthamiana*) leaf epidermal cells by *Agrobacterium*-mediated infiltration following the method described previously [85]. The fluorescent signals of GFP was visualized using a confocal laser scanning microscope (Nikon, A1, Tokyo, Japan) after 48 h of infiltration.

### 4.7. Phylogenetic Analysis of YE1

The 25 YE1-related protein sequences from other land plant species were retrieved from Phytozome database (www.phytozome.net, accessed on 06-09-2018) by using BLASTP tool with the full-length amino acids sequence of YE1 as a query and default parameters. A total of 26 protein sequences (including YE1) were aligned using ClustalW [86]. The phylogenetic tree was generated using the software program MEGA 5.0 with the neighbor-joint method [87]. Tree topology was analyzed by using full removal mode with 1000 bootstrap replications.

## 5. Conclusions

In summary, we isolated a mutant *ye1* from EMS-induced *japonica* rice mutant library. Morphological, physiological, and cellular analysis indicate that this mutant not only had yellow leaves with impaired Chl biogenesis and photosynthesis, but also exhibited an early flowering phenotype without photoperiodic-sensitivity. Map-based cloning analysis revealed that the casual gene *YE1* of this mutant was a new allele of *SE5*/*YGL2*, which encodes a chloroplast-located HO in rice. Furthermore, expression analysis uncovered that transcripts of *YE1* was induced by light, and had a diurnal-rhythmic pattern. Taken together, these results suggest that *YE1* may participates in light-dependent Chl synthesis during de-etiolation and be required for the photoperiodic flowering control simultaneously in rice.

## Figures and Tables

**Table 1:** Chl fluorescence and photosynthetic parameters of WT and *yel* mutant.

Plant	ΦPSII	ETR	F_v_/F_m_	Pn (μmol·CO_2_·m^−2^·s^−1^)	Gs (mol·H_2_O·m^−2^·s^−1^)	Ci (μmol·CO_2_·m^−2^·s^−1^)	Tr (mmol·H_2_O·m^−2^·s^−1^)
WT	0.74 ± 0.02	9.43 ± 0.05	0.84 ± 0.01	17.57 ± 0.21	0.90 ± 0.16	268.0 ± 6.55	10.62 ± 0.36
*yel*	0.71 ± 0.01 *	6.93 ± 0.07 **	0.73 ± 0.01 **	5.65 ± 0.17 **	0.48 ± 0.09 **	264.0 ± 13.50 ^NS^	3.40 ± 0.22 **

ΦPSII, effective quantum yield of PSII; ETR, electron transport rate; F_v_/F_m_, maximum quantum yield of PSII; Pn, net photosynthetic rate; Gs, stomatal conductance; Ci, intercellular CO_2_ concentration; Tr, transpiration rate. Values are given as means ± SD (*n* = 3). NS, no significant change. * (*p *< 0.05) and ** (*p *< 0.01) indicate significant differences between WT and *ye1* (Student’s *t*-test).

## Supplementary Materials

Supplementary materials can be found at http://www.mdpi.com/1422-0067/20/3/758/s1.
