# Supplementary material for: Map-Based Cloning and Functional Analysis of YE1 in Rice, Which Is Involved in Light-Dependent Chlorophyll Biogenesis and Photoperiodic Flowering Pathway
Source: Int J Mol Sci. 2019 Feb 11;20(3):758. doi: 10.3390/ijms20030758 (PMC6387406; doi:10.3390/ijms20030758)
Supplement: Supplementary file 1 [file ijms-20-00758-s001.pdf]

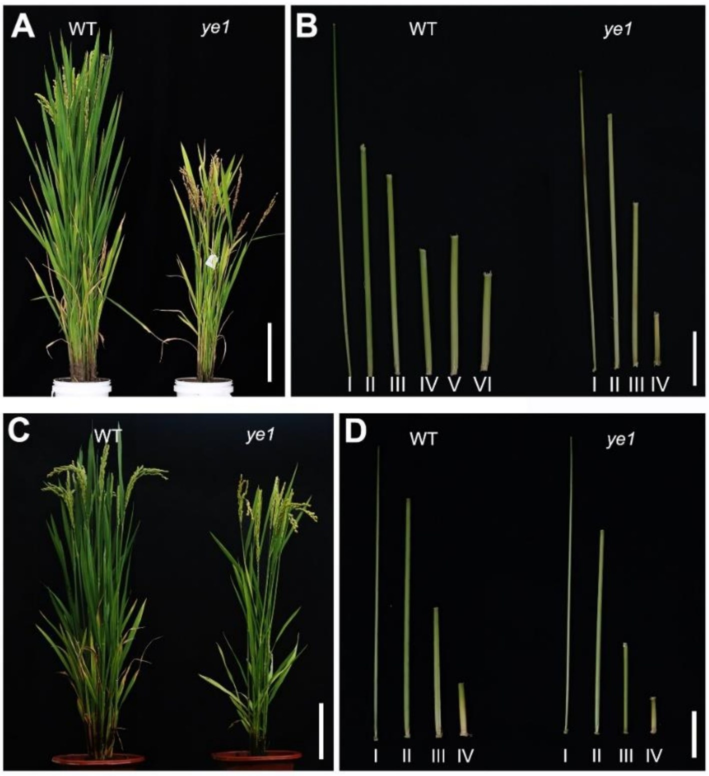

Figure S1. Phenotypes of WT and *ye1* under NLDs and NSDs. (**A**) Whole plant morphology of WT and *ye1* under NLDs at mature stage. Bar=25 cm. (**B**) Internodes of WT and *ye1* under NLDs at mature stage. Bar=5 cm. (**C**) Whole plant morphology of WT and *ye1* under NSDs at mature stage. Bar=20 cm. (**D**) Internodes of WT and *ye1* under NLDs at mature stage. Bar=5 cm.

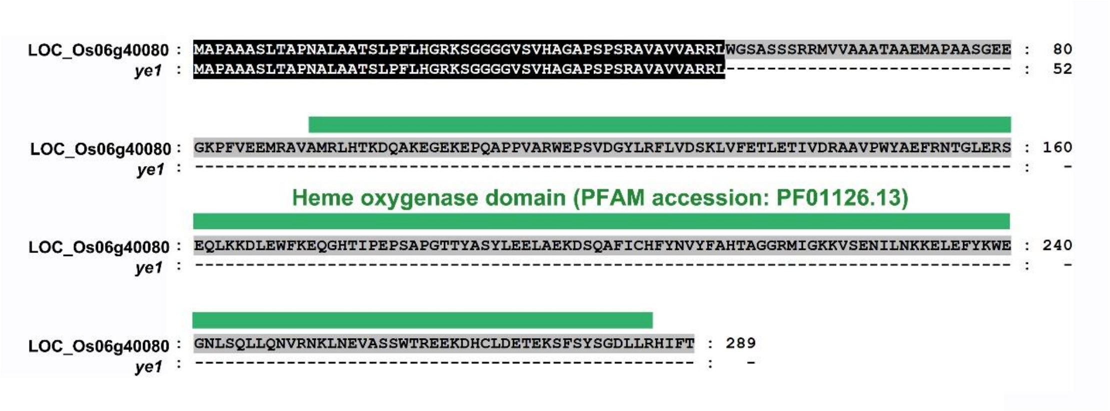

Figure S2. Peptides alignment of YE1 protein in WT (LOC_Os06g40080) and *ye1*. Green boxes indicate the region of heme oxygenase domain (PFAM accession: PF01126.13).

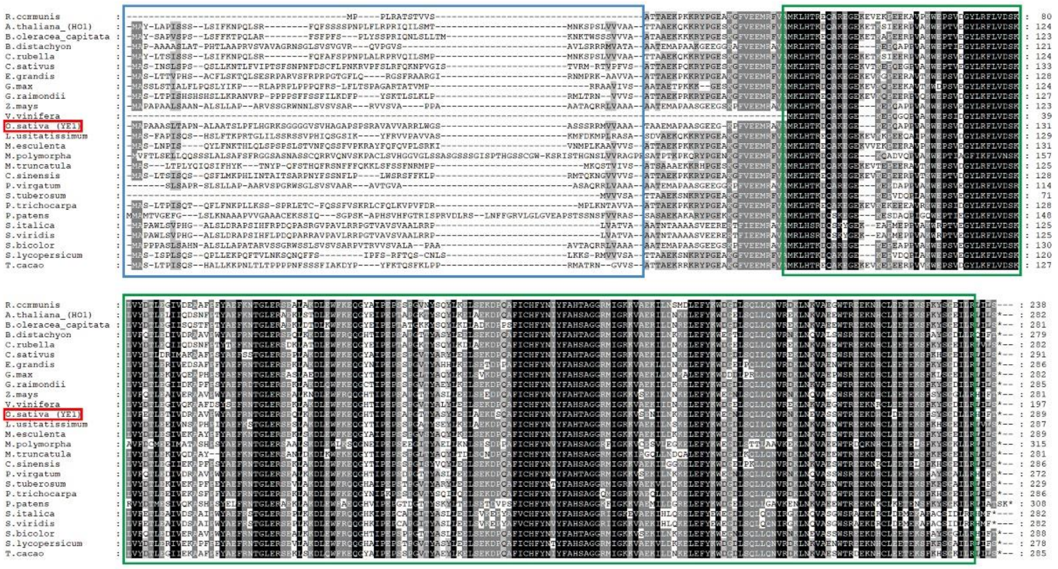

Figure S3. Peptides alignment of YE1-related proteins. Red frames indicate the YE1 protein. Blue frame indicate the high sequence diversity region in N-terminus. Green frames indicate the conserved heme oxygenase domain in C-terminus.

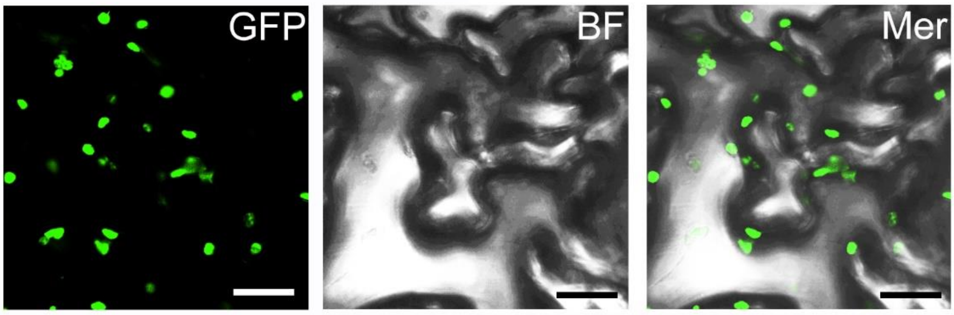

Figure S4. Subcellular location of YE1 protein in tobacco leaf epidermal cells. Bars=10 μm. BF, bright field; Mer, merge of each channel.

Table S1. Phenotype and genotype association analyses in F2 generation of *ye1*/WT.

Phenotype	No. of plants examined	No. of plants with +/+	No. of plants with +/–	No. of plants with –/–
Normal growth	47	18	29	0
Yellow leaf and early flowering	13	0	0	13

All these plants are in *japonica* wuyungeng 21 background. +/+, +/–, and –/– indicate homozygous wild type genotype, heterozygous genotype, and homozygous mutation, respectively. No., numbers.

Table S2. Information of YE1-related proteins retrieved from Phytozome.

Species	Protein Name	Description
M. Polymorpha	Mapoly0016s0114	Heme Oxygenase
S. Lycopersicum	Solyc12g009470.1	Heme Oxygenase (Biliverdin-Producing) / Heme Oxygenase (Decyclizing)
G. Max	Glyma.04g147700	Heme Oxygenase
A. Thaliana	At2g26670 (HO1)	Plant Haem Oxygenase (Decyclizing) Family Protein
E. Grandis	Eucgr.C01176	Heme Oxygenase
C. Rubella	Carubv10023804m.G	Heme Oxygenase
G. Raimondii	Gorai.010g063800	Heme Oxygenase
B. Oleracea Capitata	Bol027145	Heme Oxygenase
C. Sinensis	Orange1.1g023201m.G	Heme Oxygenase
S. Tuberosum	Pgsc0003dmg400002891	Heme Oxygenase 1
S. Viridis	Sevir.4g233900	Heme Oxygenase
O. Sativa	Loc_Os06g40080 (YE1)	Heme Oxygenase 1, Putative, Expressed
S. Italica	Seita.4g223100	Heme Oxygenase
S. Bicolor	Sobic.010g184600	Heme Oxygenase
P. Trichocarpa	Potri.018g131700	Heme Oxygenase (Biliverdin-Producing) / Heme Oxygenase (Decyclizing)
P. Patens	Pp3c3_21750	Heme Oxygenase
R. Communis	28320.T000060	Conserved Hypothetical Protein
V. Vinifera	Gsvivg01015535001	Heme Oxygenase (Biliverdin-Producing) / Heme Oxygenase (Decyclizing)
B. Distachyon	Bradi1g36640	Heme Oxygenase
M. Esculenta	Manes.14g132400	Heme Oxygenase (Biliverdin-Producing) / Heme Oxygenase (Decyclizing)
M. Truncatula	Medtr8g019320	Heme Oxygenase
C. Sativus	Cucsa.292760	Heme Oxygenase
Z. Mays	Grmzm2g043277	Heme Oxygenase
L. Usitatissimum	Lus10030092.G	Heme Oxygenase
P. Virgatum	Pavir.Da00541	Heme Oxygenase
T. Cacao	Thecc1eg038078	Plant Heme Oxygenase Family Protein

Table S3. Primers used in this study.

Primer name	Forward	Reverse	Application
RM20223	CACTTAGGAGCCGTCATGTACACC	GCTCTTGTACATGTTGAGTGTTGACC	Gene mapping
RM5371	GGCTAGCTTTAGCTGCGTTG	ACCCAGATCGAAACAACTGC	Gene mapping
ID1	CCCAATTCAAGATCCATTTCA	TTCACCCTCTTGCTGTACCC	Gene mapping
ID2	GGACCACCAAAAGGAGACTG	TACGGCTGGCTTGAGAGAAT	Gene mapping
ID3	AAGGACCACCCGTACACTTT	GATGGCCAACATGCATACTCT	Gene mapping
ID4	GGACCGAGATGAGTTGGCTA	GTGAGGACGGAGAGACAAGG	Gene mapping
ID5	CCAAAAGGACTGGGGACTCT	CAAGGGGGTGACCAAACTAA	Gene mapping
ID6	GTGGTGATCACGGAGTTTCC	TTACGAAAACCTGAAATTTTGTG	Gene mapping
YE1-SEQ	TCACTCCGCAGAAGCAAC	ATGGCACTGGTCTTATGGTC	Mutation site sequencing
YE1-COM	acgacggccagtgccaagctt ATGGCGCCCGCGGCAGC	gacctgcaggcatgcaagctt TTAGGTGAATATGTGACGGAGGAG	Complementary analysis
PYE1-COM	tgcctgcaggtcgactctaga TGCCACTGCACTAACCTTTGC	ggtacccggggatcctctaga GAGTCCCGCCCACCTCTCT	Complementary analysis
YE1-GFP	gagaacacgggggactctaga ATGGCGCCCGCGGCAGC	accacccggggatcctctaga GGTGAATATGTGACGGAGGAG	Subcellular location
Q-OsACTIN1	CTAAGCCAAGAGGAGCTGTTAT	ATAACAGATAGGCCGGTTGAAA	Expression analysis
Q-YE1	ATGTGTATTTTGCTCATACGGC	ATCTCCAGAATACGAGAACGAC	Expression analysis
Q-psbA	GCGGTTCCCTATTCAGTGCTATG	TAACCATGAGCGGCCACAATATT	Expression analysis
Q-psaA	TGGGGTTGATCCTAAGGAGATACCA	CCTCCGCGAAAACTAAGAAATTCTG	Expression analysis
Q-rbcS	CATGTGCTGTACCACTTCTTTC	AGGTACATAGAACACCTGCAAA	Expression analysis
Q-rbcL	CGTTTTGTCTTTTGTGCCAAAG	CTAATTCCCTCGCAAATACAGC	Expression analysis
Q-DVR	GTTCTTCGAGAGGGTGATCAG	GGCGTCTGAAACATTAAGAACA	Expression analysis
Q-CAO1	CATCACGGGATGCAATTGTAAT	ACATGCGATCGACTTTTACATG	Expression analysis
Q-CHLD	ATGCTTCAGACGCTGTTATTTC	AATTGGTTATCACAGCCGAAAC	Expression analysis
Q-CHLH	CGCCAAAATTATTGTAGACCGT	CCAATGACTCACCATAGGTCTT	Expression analysis
Q-PORA	TAATTTCTTCGGGGTTTAGGGG	GTAAGCCTGTGGTTTTATTGCA	Expression analysis
Q-YGL1	GGAAGATGCAGATTGATGGTTC	CCAAAAGAGCGCTAATACACTC	Expression analysis
Q-DTH7	TAGTTGATAGCGATGACTCCAC	CATTTTCAGCAGGGATGACTTC	Expression analysis

Q-DTH8	GTAACTTGGCGTTGGTTATCAG	CTCGATAACGACAACAGCATG	Expression analysis
Q-EF7	AATGTAGAGAAGAGGTTTGCCA	GCAATGGCTTTACATAGCGTTA	Expression analysis
Q-EHD2	AGCAGAAAATCGAAACTAGTGC	ATCCAGAGTCGCAAAAGTTAGA	Expression analysis
Q-HD1	TCTCAGGTCCCTCGCTTCA	GCATACGCCTTTCTTGTTTCATA	Expression analysis
Q-HD16	GAAGTTATTGCTAGAAAGGCGG	CCACCTTTACCCAACTTCCTAT	Expression analysis
Q-OsGI	GGTGGCTATGTATCATCGTACA	CAACAATGGCAAGTATAGGCTC	Expression analysis

Adaptors for plasmids construction are in lower case.
